# A Systematically Combined Genotype and Functional Combination Analysis of *CYP2E1*, *CYP2D6*, *CYP2C9*, *CYP2C19* in Different Geographic Areas of Mainland China – A Basis for Personalized Therapy

**DOI:** 10.1371/journal.pone.0071934

**Published:** 2013-10-02

**Authors:** Zhenqiang Wu, Xiaoqing Zhang, Lu Shen, Yuyu Xiong, Xi Wu, Ran Huo, Zhiyun Wei, Lei Cai, Guoyang Qi, Qingqing Xu, Daxiang Cui, Donghong Cui, Gengchun Zhao, Lin He, Shengying Qin

**Affiliations:** 1 Bio-X Institutes, Key Laboratory for the Genetics of Developmental and Neuropsychiatric Disorders (Ministry of Education), Shanghai Jiao Tong University, Shanghai, China; 2 Shanghai geno mePilot Institutes for Genomics and Human Health, Shanghai, China; 3 Institutes of Biomedical Sciences, Fudan University, Shanghai, China; 4 Department of Pharmacy, Shanghai pulmonary Hospital, Tongji University School of Medicine, Shanghai, China; 5 Wuxi Mental Health Center , Wuxi, Jiangsu, PR China; 6 Research Institute of Micro/Nano Science and Technology, Shanghai Jiao Tong University, Shanghai, China; 7 Shanghai Institute of Mental Health, Shanghai, China; 8 School of Life Sciences and Biotechnology, Shanghai Jiao Tong University, Shanghai, China; University of Tampere, Finland

## Abstract

The cytochrome P450 is the major enzyme involved in drug metabolism. Single *CYP* genotypes and metabolic phenotypes have been widely studied, but no combination analysis has been conducted in the context of specific populations and geographical areas. This study is the first to systematically analyze the combined genotypes and functional combinations of 400 samples of major *CYP* genes—*CYP2E1*, *CYP2D6*, *CYP2C9*, and *CYP2C19* in four geographical areas of mainland China. 167 different genotype combinations were identified, of which 25 had a greater than 1% frequency in the Chinese Han population. In addition, phenotypes of the four genes for each sample were in line with the predictions of previous studies of the four geographical areas. On the basis of the genotype classification, we were able to produce a systemic functional combinations analysis for the population. 25 of the combinations detected had at least two non-wild phenotypes and four showed a frequency above 1%. A bioinformatics analysis of the relationship between particular drugs and multi-genes was conducted. This is the first systematic study to analyze genotype combinations and functional combinations across whole Chinese population and could make a significant contribution in the field of personalized medicine and therapy.

## Introduction

It is well established that individual patients can have significantly different responses to clinical drugs. Drug concentration in plasma can vary ~600-fold between two individuals of the same weight who have received the same drug dosage, and this can result in non-efficiency or adverse drug reactions (ADRs) [1]. ADR ranks as the 5th leading cause of death and illness in the developed world, imposing costs estimated at 100 billion USD in the US and causing over 100,000 deaths every year [2,3]. Because of its large population and poor medical conditions, the problem of ADR fatality is even more serious in China. A report by the WHO estimated that 2.5 million Chinese patients are hospitalized annually due to ADR, of whom 190,000 lose their lives [4]. Within the pathway of drug response, it is well known that the cytochrome P450 (*CYP*) superfamily plays a critical role in metabolic biotransformation, mediating up to 90% of all drug oxidation metabolism [5]. Five major *CYP* genes—*CYP2E1*, *CYP2D6*, *CYP2C9* ,*CYP2C19* and *CYP3A4/5* play the most important role in drug metabolism, respectively accounting for 4%, 19%, 16%, 8% and 34% of the metabolizing process [6]. Different enzymes within the *CYP* family metabolize different drugs and many drugs are metabolized by a combination of *CYP*s.

Individual variability in drug response can be attributable to factors such as age, gender, or environmental factors but genetic differences in particular, can account for 15%–30% ,or even higher in some groups, of inter-individual differences in drug metabolism and response [1]. Polymorphisms of the *CYP* enzymes have been widely identified, with two or more variant alleles. These variants in the DNA sequence of genes, to some extent, decrease, increase or completely abolish the enzyme activity. Individuals can be classified as extensive metabolizers (EM or wild type), poor metabolizers (PM), intermediate metabolizers (IM) and ultrarapid metabolizers (UM) according to their ability to metabolize drug substrates.

The enzyme activity variability of *CYP* genes attributable to genetic factors can be used as a predictor for individualized therapy to improve clinical efficacy or avoid ADR. The relationship between specific *CYP* enzyme activity and its gene polymorphism has been widely studied (http://www.CYPalleles.ki.se/). It has been shown that allele frequencies vary largely between different populations and geographic areas and a number of pharmacogenomics studies have investigated different drug metabolism genes in specific geographic areas and ethnic groups. Our own group has also conducted a gene polymorphism analysis of different *CYP* genes in the Chinese population [7–10]. However, most of these studies have focused on single genes [11], drug metabolism usually involves multiple *CYP* genes. Multi-gene analysis is therefore important in drug response evaluation but, to date, no systematic combined genotype and functional combinations analysis of multiple *CYP* genes in different geographic areas for the same population has been undertaken. In the present study, we focused on analyzing the functional combinations of four major *CYP* genes—*CYP2E1*, *CYP2D6*, *CYP2C9* and *CYP2C19* genes in different geographic areas in within the Chinese Han population. *CYP*3A4/5 was not among the genes studied since its enzyme variation is associated more with non-genetic factors than directly with genetic factors. Our work is the first to apply a systemic combined analysis to the field of personalized drug provision and could provide a useful basis for clinical genotype testing.

## Materials and Methods

### Subjects

The samples for the study were collected from 400 healthy unrelated volunteers living in four different areas of the Chinese mainland. 100 subjects from Xi’an City, which lies in the west of China; 100 subjects from Shanghai City, which lies in the east of China; 100 subjects from Shenyang City, which lies in the north of China; and 100 subjects from Shantou City, which lies in the south of China. Each group of 100 subjects consisted of 50 males and 50 females between 18 and 53 years of age. All subjects were judged to be in good health in terms of their medical history and after a physical examination. All the volunteers in this study were of homogenous Chinese Han ethnicity. The study was approved by the Shanghai Ethical Committee of Human Genetic Resources and all subjects gave informed consent for their participation.

### Polymerase chain reaction condition and DNA sequencing

Systematic polymorphism screening had been performed using long-PCRs and direct sequencing in some of our previous work. Genomic DNA was isolated from peripheral blood using standard procedures [12]. PCR primers were designed to amplify 2000 bp of the 5´-flanking regions and all exons of the *CYP* gene. In case any sequence was missed, overlapping primers were used. The PCRs were carried out on the Gene Amp® PCR system 9700 (Applied Biosystems, CA, USA). The amplification mixture contained a final volume of 25µl: 10 ng of genomic DNA, 10 mM Tris-HCl (pH8.3), 50 mM KCl, 1.5–3.0 mM MgCl2, 200 mM dNTP, 1 mM of each primer and 0.25 U Taq DNA polymerase. The amplification conditions were: 95°C for 1 min, followed by 30–35 cycles at 95°C for 30 s, 50–65°C for 1 min, 72°C for 1 min, then a final extension at 72°C for 10 min. Preparation of DNA for sequencing included incubation of PCR products with 0.1 U of shrimp alkaline phosphatase (Roche, Basel, Switzerland) and 0.5 U of exonulease I (New England Biolabs Inc., MA, USA) at 37°C for 45 min, followed by heat inactivation at 85°C for 20 min. The PCR products were sequenced using an ABI Prism® BigDye Terminator Cycle Sequencing Kit, version 3.1 (Applied Biosystems) on an ABI Prism 3730 sequencer.

### Combined genotype analysis

The analysis of allele and genotype frequencies of *CYP2E1*, *CYP2D6*, *CYP2C9*, and *CYP2C19* in the Chinese Han population had been performed in one of our earlier studies. Genotypes of the four genes were reviewed again for each sample and rearranged as necessary for the current investigation (http://www.CYPalleles.ki.se/). On the basis of each sample data, a combined genotype analysis (Table 1) was performed, calculating the combination frequency of the four CYP genes found in the sample. The comparison of combined genotype frequencies among different geographic of chinese have been done usingχ^2^ tests with a significant level set at 0.05.

**Table 1:** The combined genotype frequency in four different geographical Chinese populations.

**Combined genotype**				**Combined genotype frequency**					
***CYP2E1***	***CYP2D6***	***CYP2C9***	***CYP2C19***	**Shanghai**	**Xi’an**	**Shenyang**	**Shantou**	**Chinese**	**p value^a^**
*1/*1	*1/*1	*1/*1	*1/*1	3.13%	5.21%	0	1.04%	2.34%	0.264
*1/*1	*1/*1	*1/*1	*1/*2	3.13%	1.04%	3.13%	0	1.82%	0.059
*1/*1	*1/*10	*1/*1	*1/*1	4.17%	7.29%	9.38%	5.21%	6.51%	0.501
*1/*1	*1/*10	*1/*1	*1/*2	2.08%	2.08%	2.08%	3.13%	2.34%	0.317
*1/*1	*1/*10	*1/*3	*1/*1	4.17%	0	0	0	1.04%	0.79
*1/*1	*1/*2	*1/*1	*1/*1	1.04%	2.08%	1.04%	0	1.04%	0.18
*1/*1	*10/*10	*1/*1	*1/*1	2.08%	4.17%	5.21%	4.17%	3.91%	0.165
*1/*1	*10/*10	*1/*1	*1/*2	2.08%	2.08%	1.04%	7.29%	3.13%	0.105
*1/*1	*10/*10	*1/*1	*2/*2	2.08%	2.08%	0	1.04%	1.30%	0.18
*1/*1	*2/*10	*1/*1	*1/*1	1.04%	1.04%	2.08%	0	1.04%	0.819
*1/*7	*1/*1	*1/*1	*1/*1	1.04%	0	3.13%	5.21%	2.34%	0.264
*1/*7	*1/*1	*1/*1	*1/*2	0	3.13%	0	3.13%	1.56%	0.434
*1/*7	*1/*10	*1/*1	*1/*1	0	2.08%	5.21%	2.08%	2.34%	0.739
*1/*7	*1/*10	*1/*1	*1/*2	1.04%	4.17%	2.08%	0	1.82%	0.368
*1/*7	*10/*41	*1/*1	*1/*1	3.13%	0	1.04%	0	1.04%	0.317
*1/*7	*10/*10	*1/*1	*1/*1	1.04%	1.04%	4.17%	5.21%	2.86%	0.529
*1/*7	*10/*10	*1/*1	*1/*2	0	3.13%	0	1.04%	1.04%	0.317
*5/*7	*1/*1	*1/*1	*1/*1	3.13%	0	1.04%	1.04%	1.30%	0.655
*5/*7	*1/*10	*1/*1	*1/*1	4.17%	2.08%	5.21%	4.17%	3.91%	0.165
*5/*7	*1/*10	*1/*1	*1/*2	3.13%	3.13%	1.04%	1.04%	2.08%	0.157
*5/*7	*10/*10	*1/*1	*1/*1	1.04%	1.04%	3.13%	4.17%	2.34%	0.717
*5/*7	*10/*10	*1/*1	*1/*2	3.13%	1.04%	0	3.13%	1.82%	0.059
*7/*7	*1/*10	*1/*1	*1/*1	1.04%	1.04%	2.08%	2.08%	1.56%	0.414
*7/*7	*10/*10	*1/*1	*1/*1	1.04%	2.08%	2.08%	2.08%	1.82%	0.059
*7/*7	*10/*10	*1/*1	*1/*2	1.04%	0	0	3.13%	1.04%	0.317

a The P value is for the comparison of the combined genotype frequencies among the fourdifferent geographic populations

### Functional combinations analysis

Our previous studies had established the predicted metabolic phenotypes (EM, IM, PM, UM) of the four CYP genes in different geographic areas based on genotype results for each sample. Individuals with two alleles coding for “normal” enzyme function were termed EM, whereas those with two variant alleles resulting in an inactive or absent enzyme were termed PM [13]. If one of alleles was normal but another resulted in reduced enzyme activity, it was regarded as IM. In some rare cases, such as for the *CYP2D6* gene in some populations, gene duplication and multiplication can lead to UM. A functional combinations analysis of the four CYP genes was completed by aggregating each sample’s combined metabolic phenotypes and calculating the functional combinations frequency for the overall population. The comparison of functional combinations frequencies among different geographic of chinese have been done usingχ^2^ tests with a significant level set at 0.05. All tastics were been implemented on SPSS 17.0 platform.

### Pharmacogenomics Associations analysis

The well-known pharmacogenomics associations have been summarized, including four CYP genes used in this study and other genes and polymorphisms related to drug response drawn from various websites (http://www.pharmgkb.org/search/knownPairs.action; http://stitch.embl.de/)

## Results

The genotype analysis detected a total of 167 genotype combinations (Table S1) of the four genes in the sample of 400 Chinese Han subjects. Most of these combinations appeared in less than 1% of all samples. 25 combinations with frequencies greater than 1% were used as a focus group, making up 53.39% of all samples (Table 1). The genotype of *CYP2C9* plays a small part in the focus group, featuring almost entirely as wild type *1/*1 with only one as type *1/*3. The most common 3 allele combinations (the unified order for combinations referred to later in this article) are (*CYP2E1*-*CYP2D6*-*CYP2C9*-*CYP2C19*) (*1/1, *1/*10, *1/*1, *1/*1), (*1/1, *10/*10, *1/*1, *1/*1), (*5/7, *1/*10, *1/*1, *1/*1), respectively, and these appear in 6.51%, 3.91% and 3.91% of the Chinese population. Of the 25 genotype combinations, 11 exceeded a 2% frequency in the population, accounting for a 34.11% frequency overall. None of the combinations exhibit the obvious differences between four geographic areas in chinese (P value>0.05).

On the basis of previous genotype analyses, we identified all the metabolic phenotypes for the four CYP genes in the four areas and in China as a whole (Table 2). The EM for *CYP2D6* (60.42%) and *CYP2C19* (53.39%) is significantly lower than for *CYP2E1* (93.49%) and *CYP2C9* (88.02%) in the Chinese Han population. The distribution of intermediate metabolizer phenotypes for *CYP2D6* and *CYP2C19* together account for more than 30%, in contrast to *CYP2E1* (2.60%) and *CYP2C9* (9.11%). The ultrarapid metabolizer (UM) phenotype did not feature for *CYP2E1* and *CYP2C9*, and displayed only low frequencies for *CYP2D6* (0.78%) and *CYP2C19* (1.30%). Comparing the incidence of poor metabolizers (PM) among the four CYP phenotype frequencies, *CYP2C19* registered at 11.20% compared with fewer than 4% for all the others. The differences between four geographic areas were clear for PM of *CYP2E1*, IM of CYP2C9 and CYP2C19, EM of *CYP2C19*(P value<0.05).

**Table 2:** The phenotype frequency in four different geographical Chinese populations.

	**Metabolic phenotype frequency**					
**CYP 2E1 metabolic phenotype**	**Shanghai**	**Xi’an**	**Shenyang**	**Shantou**	**Chinese**	**p value^b^**
(**U**)ultrarapid metabolizer	0	0	0	0	0	/
(**E**)Extensitive metabolizer	86.46%	96.88%	97.92%	92.71%	93.49%	0.813
(**I**)Inermediate metabolizer	4.17%	1.04%	1.04%	4.17%	2.60%	0.058
(**P**)Poor metabolizer	9.38%	2.08%	1.04%	3.13%	3.91%	0.016
**CYP 2D6 phenotype**						
(**U**)ultrarapid metabolizer	1.04%	0	1.04%	1.04%	0.78%	0.655
(**E**)Extensitive metabolizer	66.67%	61.46%	63.54%	50.00%	60.42%	0.436
(**I**)Inermediate metabolizer	32.29%	37.50%	35.42%	47.92%	38.28%	0.286
(**P**)Poor metabolizer	0	1.04%	0	1.04%	0.52%	0.343
**CYP 2C9 phenotype**						
(**U**)ultrarapid metabolizer	0	0	0	0	0	/
(**E**)Extensitive metabolizer	83.33%	92.71%	79.17%	96.88%	88.02%	0.492
(**I**)Inermediate metabolizer	14.58%	5.21%	14.58%	2.08%	9.11%	0.001
(**P**)Poor metabolizer	2.08%	2.08%	1.04%	0	1.30%	0.18
**CYP 2C19 phenotype**						
(**U**)ultrarapid metabolizer	0	1.04%	2.08%	2.08%	1.30%	0.18
(**E**)Extensitive metabolizer	44.79%	43.75%	72.92%	52.08%	53.39%	0.017
(**I**)Inermediate metabolizer	38.54%	39.58%	17.71%	36.46%	33.07%	0.022
(**P**)Poor metabolizer	15.63%	14.58%	6.25%	8.33%	11.20%	0.084

b The P value is for the comparison of the metabolic phenotype frequencies among the fourdifferent geographic populations.

By integrating the results of combined genotype analysis and metabolic phenotype predictions, we combined the metabolic phenotypes of four genes and analyzed the functional combinations frequency in the four geographical areas of China. We choose 25 functional combinations which included more than two non-wild types (UM, IM, PM) of the four genes (Table 3). All these groups were classified into 9 types, 6 containing two non-wild phenotypes (such as IM-IM-EM-EM) and the remaining combinations containing three non-EM phenotypes (such as PM-IM-EM-PM). Four functional combinations were identified as having greater than 1% frequency in the Chinese population. The two most important of these consist of non-EMs of both *CYP2D6* and *CYP2C19*, accounting for 10.94% (EM-IM-EM-IM) and 4.69% (EM-IM-EM-PM) respectively. For the majority of polymorphisms of *CYP2D6* and its functional variations which are obviously greater than other three genes, the frequency of non-wild combinations with *CYP2C9* (16.93%) and *CYP2C19* (2.86%) are higher compared with other combination which have a frequency of less than 2%. Only one type of combination (EM-IM-EM-IM) shows obvious differences betweeen four geographic areas(P value<0.01).

**Table 3:** The functional combinations frequency of four *CYP* genes in four different geographical populations.

**Functional combinations**				**Functional combinations frequency**					
**CYP2E1**	**CYP2D6**	**CYP2C9**	**CYP2C19**	**Shanghai**	**Xi’an**	**Shenyang**	**Shantou**	**Chinese**	**p value^c^**
IM	IM	EM	EM	0	0	1.04%	1.04%	0.52%	0.825
PM	IM	EM	EM	0	0	0	2.08%	0.52%	0.652
Total* CYP2E1 & CYP2D6				0	0	1.04%	3.12%	1.04%	0.317
IM	EM	PM	EM	0	1.04%	0	0	0.26%	0.716
PM	EM	IM	EM	1.04%	0	0	0	0.26%	0.645
Total* CYP2E1 & CYP2C9				1.04%	1.04%	0	0	0.52%	0.825
IM	EM	EM	IM	1.04%	0	0	0	0.26%	0.745
PM	EM	EM	IM	2.08%	1.04%	0	0	0.78%	0.654
PM	EM	EM	PM	3.12%	0	0	1.04%	0.26%	0.463
Total* CYP2E1 & CYP2C19				4.16%	1.04%	0	1.04%	0.43%	0.414
EM	IM	IM	EM	2.08%	1.04%	5.21%	1.04%	2.34%	0.368
EM	IM	PM	EM	0	1.04%	1.04%	0	0.52%	0.364
Total* CYP2D6 & CYP2C9				2.08%	2.08%	6.25%	1.04%	2.86%	0.178
EM	IM	EM	IM	12.50%	9.38%	3.13%	18.75%	10.94%	0.006
EM	IM	EM	PM	8.33%	5.21%	0	5.21%	4.69%	0.637
EM	IM	EM	UM	1.04%	1.04%	0	1.04%	0.52%	0.541
EM	PM	EM	IM	0	0	0	1.04%	0.26%	0.642
EM	UM	EM	IM	0	0	0	1.04%	0.52%	0.476
Total* CYP2D6 & CYP2C19				9.37%	15.63%	3.13%	27.08%	16.93%	0.001
EM	EM	IM	IM	1.04%	1.04%	2.08%	0	1.04%	0.522
EM	EM	IM	PM	0	1.04%	0	0	0.26%	0.742
EM	EM	PM	IM	2.08%	0	0	0	0.52%	0.655
Total* CYP2C9 & CYP2C19				3.12%	2.08%	2.08%	0	1.82%	0.714
EM	IM	IM	IM	1.04%	0	0	0	0.26%	0.754
EM	IM	IM	PM	1.04%	0	0	0	0.26%	0.623
EM	IM	IM	UM	0	0	1.04%	0	0.26%	0.844
EM	UM	IM	IM	0	0	1.04%	0	0.26%	0.423
Total* CYP2D6 & CYP2C9 & CYP2C19				2.08%	0	2.08%	0	1.04%	0.765
IM	EM	IM	IM	1.04%	0	0	0	0.26%	0.645
Total* CYP2E1 & CYP2C9 & CYP2C19				1.04%	0	0	0	0.26%	0.645
IM	IM	EM	PM	1.04%	0	0	1.04%	0.52%	0.324
PM	IM	EM	IM	2.08%	1.04%	0	0	0.78%	0.564
PM	IM	EM	PM	1.04%	0	1.04%	0	0.52%	0.825
Total* CYP2E1 & CYP2D6 & CYP2C19				4.16%	1.04%	1.04%	1.04%	1.82%	0.705

c The P value is for the comparison of the functional combinations frequencies among the fourdifferent geographic populations

* Total *CYP* stands for non-EM (wild) phenotype in combination

**Figure 1:**
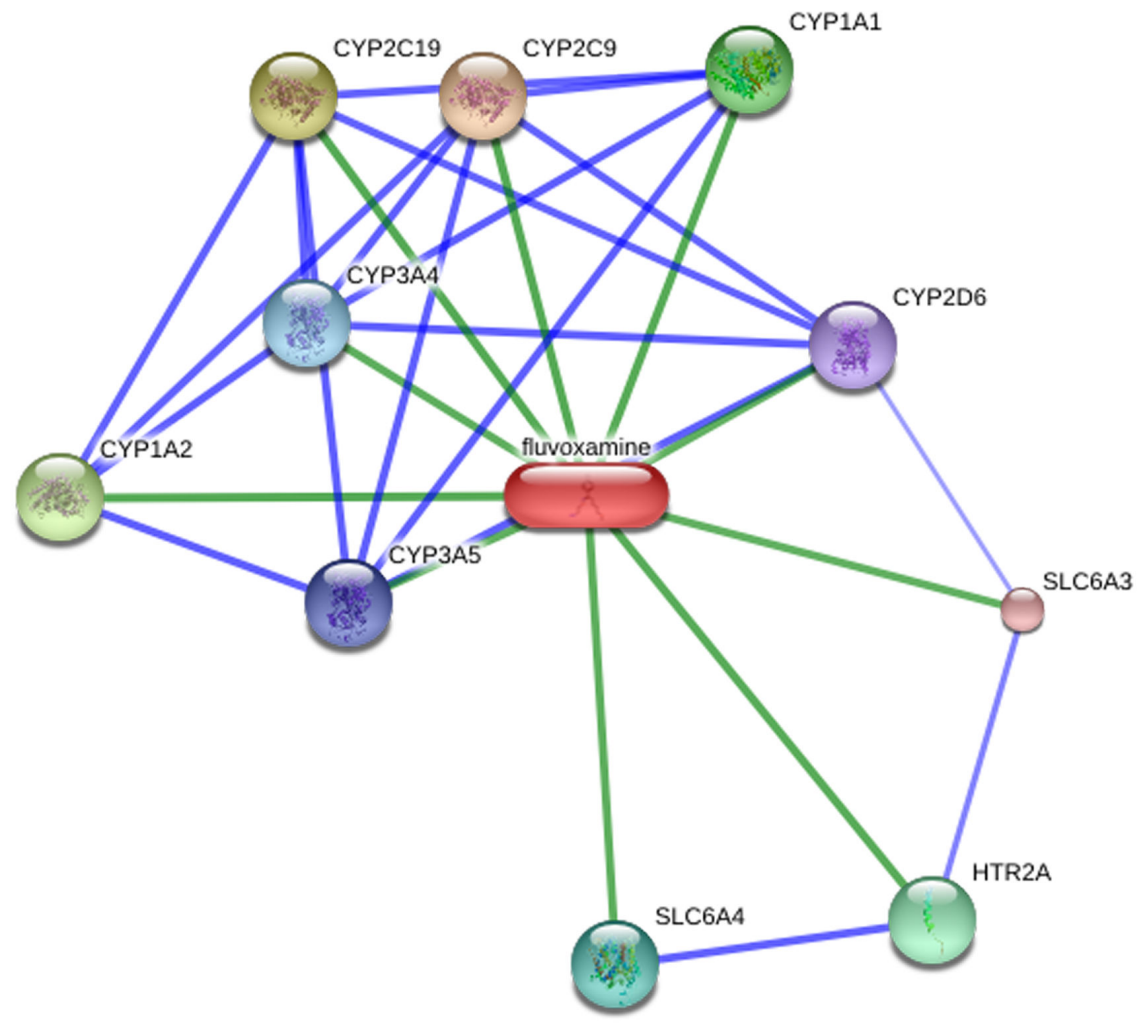
Drug-gene relationship network centered on fluvoxamine.

In order to demonstrate the importance of combined genotype and functional combinations analysis, we investigated previous research into well-known pharmacogenomics association between drugs and genes and details of several drugs that are metabolized by more than one CYP genes (Table 4). We also used bioinformatics (STICH 3.1) analysis to investigate the concrete relationship between drugs and drug response related genes. For instance we used this method to investigate the drug response related genes for the antidepressant—fluvoxamine and the results are shown in Figure 1. This drug has complex associations with 7 metabolizing genes and 3 other related genes.

**Table 4:** Relationships between drugs and drug related genes.

**Drug**	**Drug related genes**	**Drug function**
Citalopram	*CYP2D6* , *CYP2C19*	Antidepressant medication
Fluvoxamine	CYP1A2, *CYP2C19*, *CYP2C9*, *CYP2D6*, CYP3A4	Antidepressant medication
Imipramine	*CYP2D6*, *CYP2C19*	Antidepressant medication
Clopidogrel	*CYP2C19*, CYP3A4	Antiplatelet agent
Chlorzoxazone	*CYP2E1*, CYP1A2	Muscle relaxant
Modafinil	*CYP2D6*, *CYP2C19*, *CYP2C9*	Stimulant-like drug
Nelfinavir	*CYP2C19*, CYP3A	Antiretroviral drug
Phenprocoumon	*CYP2C9*, VKORC1	Antiretroviral drug
Warfarin	*CYP2C9*, VKORC1	Anticoagulant

## Discussion

Pharmacogenetics has been seen as having great promise for individualized therapy. Several studies have demonstrated that genotype testing has the potential to optimize personalized medicine use [14,15]. *CYP450* is the major enzyme involved in drug metabolism, accounting for about 75% of the total process [16]. Pharmacogenetics tests for *CYP450* genotypes have been developed and granted by FDA [17]. However, the current studies and commercial tests are mainly limited to the effects of single gene polymorphisms, despite the fact that most drugs metabolism exhibits poly-genetic traits. Our study is the first to investigate the combined genotypes and functional combinations of four main *CYP* genes in the Chinese population and could provide a theoretical basis for systematically evaluating drug efficacy in the context of personalized medicine.

The 167 genotype combinations discovered in our study provide a complete profile of the genotype variations of the main *CYP* genes in the Chinese Han population. Among the 25 principal genotype combinations based on the genotype of *CYP2C9*, almost all displayed the wild type *1/*1 with only one displaying type *1/*3, which would suggest that *CYP2C9* is particularly common in the Chinese population. Though we concentrated on genotype combinations with an incidence of more than 1%, given the large size and wide distribution of the Chinese Han population, rare combinations could also play a significant role in individual drug response. The geographical variations in genotype combinations which we observed, may not be permanent as living environments and lifestyles change over time within regions and within sub-groups of the population. However, variation in regional results may also be due to our relatively small-size samples, and the results would need to be confirmed by larger-size samples.

The metabolic phenotype analysis of the four main *CYP* genes revealed significant distributional differences of the four CYP genes across the population. Our results identified 11.2% poor metabolizers for *CYP2C19*, which is consistent with a previous report showing a frequency of 13.7% in the Chinese population [18]. Phenotype distribution differences across the four geographical areas were identified in our study, as, for example the EM and PM of *CYP2C19* for four geographic areas are significantly different(P value <0.05). Phenotype differences attributable to geographical or population variation have been identified around the world, such as the frequency of poor metabolizers for *CYP2D6* which is approximately 3-10% in Caucasians, 1–2% in Orientals and 1.9% among Afro Americans [19,20]. Our metabolic phenotype analysis effectively complements this data in terms of the Chinese Han population. Our analysis is the first to study functional combinations in four areas of the Chinese Han population, and should provide a useful reference point for effective clinical medication. As shown in Table 4, the metabolism of most drugs is always related to more than one CYP enzyme. Such as antidepressant—fluvoxamine, metabolized by both CYP2D6, CYP2C9 and CYP2C19, only one type CYP genotype analysis is not enough as genetic evidence in clinical. In addition, effective therapy for most complex diseases generally needs combined therapy rather than a mono-drug approach, involving more than one kind of drug response pathway. Furthermore, as shown in Figure 1, drug response can often be related to more complex gene relationships. Systemic functional combinations analysis will therefore become increasingly important for a precise evaluation of drug response. According to our results, the two functional combinations with the highest frequencies (10.94% and 4.69%) involve non-EMs of *CYP2D6* and *CYP2C19*, and this data could be useful for drug response evaluation for the many antidepressant drugs metabolized by these two genes as listed in Table 4.

In conclusion, in the present study we conducted a systematically combined genotype and functional combination analysis of four CYP genes in four different geographic areas of mainland China. Data on the profiles of the combined alleles and functional combinations of the four main *CYP* genes could provide a foundation for a systematic pharmacogenomics evaluation of drug efficacy in the context of individualized therapy.

## Supporting Information

Table S1 **The total 167 kinds of combined genotype frequency in four different geographical Chinese populations.**
(DOCX)
