# Supplementary material for: A Systematically Combined Genotype and Functional Combination Analysis of CYP2E1, CYP2D6, CYP2C9, CYP2C19 in Different Geographic Areas of Mainland China – A Basis for Personalized Therapy
Source: PLoS One. 2013 Oct 2;8(10):e71934. doi: 10.1371/journal.pone.0071934 (PMC3788764; doi:10.1371/journal.pone.0071934)
Supplement: Table S1 — The total 167 kinds of combined genotype frequency in four different geographical Chinese populations. (DOCX) [file pone.0071934.s001.docx]

**Table S1.** The total 167 kinds of combined genotype frequency in four different geographical Chinese populations

| **Genotype** | | | | **Combined genotype frequency(%)** | | | | |
| --- | --- | --- | --- | --- | --- | --- | --- | --- |
| **CYP2E1** | **CYP2D6** | **CYP2C9** | **CYP2C19** | **Shanghai** | **Xi'an** | **Shenyang** | **Shantou** | **Chinese** |
| *1/*1 | *1/*1 | *1/*1 | *1/*1 | 3.13% | 5.21% | 0 | 1.04% | 2.34% |
| *1/*1 | *1/*1 | *1/*1 | *1/2 | 3.13% | 1.04% | 3.13% | 0 | 1.82% |
| *1/*1 | *1/*1 | *1/*1 | *1 /*15 | 0 | 0 | 0 | 1.04% | 0.26% |
| *1/*1 | *1/*1 | *1/*1 | *2/*15 | 1.04% | 0 | 1.04% | 0 | 0.52% |
| *1/*1 | *1/*1 | *1/*1 | *2/*2 | 1.04% | 0 | 0 | 0 | 0.26% |
| *1/*1 | *1/*1 | *1/*1 | *2/*3 | 1.04% | 0 | 0 | 0 | 0.26% |
| *1/*1 | *1/*10 | *1/*1 | *1/1 | 4.17% | 7.29% | 9.38% | 5.21% | 6.51% |
| *1/*1 | *1/*10 | *1/*1 | *1/2 | 2.08% | 2.08% | 2.08% | 3.13% | 2.34% |
| *1/*1 | *1/*10 | *1/*1 | *1/*3 | 0 | 1.04% | 0 | 1.04% | 0.52% |
| *1/*1 | *1/*10 | *1/*1 | *1/*17 | 0 | 0 | 0 | 1.04% | 0.26% |
| *1/*1 | *1/*10 | *1/*1 | *2/*2 | 0 | 1.04% | 1.04% | 0 | 0.52% |
| *1/*1 | *1/*10 | *1/*1 | *3/*17 | 0 | 1.04% | 0 | 0 | 0.26% |
| *1/*1 | *1/*10 | *1/*3 | *1/1 | 4.17% | 0 | 0 | 0 | 1.04% |
| *1/*1 | *1/*10 | *1/*31 | *1/1 | 0 | 0 | 1.04% | 0 | 0.26% |
| *1/*1 | *1/*10 | *3/*31 | *1/*3 | 0 | 0 | 1.04% | 0 | 0.26% |
| *1/*1 | *1/*2 | *1/*1 | *1/1 | 1.04% | 2.08% | 1.04% | 0 | 1.04% |
| *1/*1 | *1/*2 | *1/*1 | *1/2 | 0 | 1.04% | 0 | 1.04% | 0.52% |
| *1/*1 | *1/*2 | *1/*2 | *1/1 | 0 | 0 | 1.04% | 0 | 0.26% |
| *1/*1 | *1/*2 | *1/*3 | *1/1 | 0 | 1.04% | 0 | 0 | 0.26% |
| *1/*1 | *1/*2 | *1/*8 | *1/1 | 1.04% | 0 | 0 | 0 | 0.26% |
| *1/*1 | *1/*41 | *1/*1 | *2/*15 | 0 | 0 | 0 | 1.04% | 0.26% |
| *1/*1 | *1/*41 | *1/*1 | *2/*2 | 1.04% | 0 | 0 | 0 | 0.26% |
| *1/*1 | *10/*41 | *1/*1 | *1/1 | 1.04% | 0 | 1.04% | 0 | 0.52% |
| *1/*1 | *10/*41 | *1/*1 | *1/2 | 0 | 0 | 1.04% | 0 | 0.26% |
| *1/*1 | *10/*41 | *1/*3 | *1/1 | 0 | 0 | 1.04% | 0 | 0.26% |
| *1/*1 | *10/*10 | *1/*1 | *1/1 | 2.08% | 4.17% | 5.21% | 4.17% | 3.91% |
| *1/*1 | *10/*10 | *1/*1 | *1/2 | 2.08% | 2.08% | 1.04% | 7.29% | 3.13% |
| *1/*1 | *10/*10 | *1/*1 | *2/*2 | 2.08% | 2.08% | 0 | 1.04% | 1.30% |
| *1/*1 | *10/*10 | *1/*1 | *2/*3 | 1.04% | 0 | 0 | 0 | 0.26% |
| *1/*1 | *10/*10 | *1/*3 | *1/1 | 0 | 0 | 1.04% | 0 | 0.26% |
| *1/*1 | *10/*10 | *1/*3 | *1/*3 | 1.04% | 0 | 0 | 0 | 0.26% |
| *1/*1 | *10/*10 | *1/*3 | *2/*2 | 1.04% | 0 | 0 | 0 | 0.26% |
| *1/*1 | *10/*10 | *3/*8 | *1/1 | 0 | 0 | 1.04% | 0 | 0.26% |
| *1/*1 | *2/*10 | *1/*1 | *1/1 | 1.04% | 1.04% | 2.08% | 0 | 1.04% |
| *1/*1 | *2/*10 | *1/*1 | *1/2 | 0 | 1.04% | 0 | 1.04% | 0.52% |
| *1/*1 | *2/*10 | *1/*1 | *1/*3 | 1.04% | 0 | 0 | 0 | 0.26% |
| *1/*1 | *2/*10 | *1/*2 | *1/2 | 0 | 0 | 1.04% | 0 | 0.26% |
| *1/*1 | *2/*2 | *1/*1 | *1/1 | 1.04% | 0 | 0 | 0 | 0.26% |
| *1/*1 | *2/*2 | *1/*1 | *1/*3 | 1.04% | 0 | 0 | 0 | 0.26% |
| *1/*1 | *2/*2 | *1/*8 | *1/1 | 0 | 0 | 1.04% | 0 | 0.26% |
| *1/*1 | *2/*4 | *1/*1 | *1/1 | 0 | 0 | 1.04% | 0 | 0.26% |
| *1/*1 | *2/*41 | *1/*1 | *1/2 | 1.04% | 0 | 0 | 0 | 0.26% |
| *1/*1 | *2/*5 | *1/*1 | *1/1 | 0 | 0 | 1.04% | 0 | 0.26% |
| *1/*1 | *5/*10 | *1/*1 | *1/1 | 0 | 2.08% | 0 | 0 | 0.52% |
| *1/*1 | *5/*10 | *1/*1 | *1/2 | 0 | 1.04% | 0 | 1.04% | 0.52% |
| *1/*1 | *5/*10 | *1/*1 | *2/*3 | 0 | 1.04% | 0 | 0 | 0.26% |
| *1/*1 | 1*2/10 | *1/*1 | *1/1 | 0 | 1.04% | 0 | 0 | 0.26% |
| *1/*1 | 10*2/10 | *1/*1 | *1/1 | 0 | 0 | 1.04% | 0 | 0.26% |
| *1/*5 | *1/*1 | *1/*1 | *1/1 | 0 | 0 | 0 | 1.04% | 0.26% |
| *1/*5 | *1/*1 | *1/*8 | *1/*3 | 1.04% | 0 | 0 | 0 | 0.26% |
| *1/*5 | *1/*10 | *1/*1 | *1/1 | 0 | 0 | 0 | 1.04% | 0.26% |
| *1/*5 | *1/*10 | *3/*11 | *1/1 | 0 | 1.04% | 0 | 0 | 0.26% |
| *1/*5 | *10/*10 | *1/*1 | *2A/*2A | 0 | 0 | 0 | 1.04% | 0.26% |
| *1/*5 | *2/*10 | *1/*1 | *1/2 | 1.04% | 0 | 0 | 0 | 0.26% |
| *1/*5 | *5/*10 | *1/*1 | *1/1 | 0 | 0 | 1.04% | 1.04% | 0.52% |
| *1/*7 | *1/*1 | *1/*1 | *1/1 | 1.04% | 0 | 3.13% | 5.21% | 2.34% |
| *1/*7 | *1/*1 | *1/*1 | *1/2 | 0 | 3.13% | 0 | 3.13% | 1.56% |
| *1/*7 | *1/*1 | *1/*1 | *2/*2 | 0 | 0 | 1.04% | 0 | 0.26% |
| *1/*7 | *1/*1 | *1/*31 | *2/*2 | 0 | 0 | 1.04% | 0 | 0.26% |
| *1/*7 | *1/*10 | *1/*1 | *1/1 | 0 | 2.08% | 5.21% | 2.08% | 2.34% |
| *1/*7 | *1/*10 | *1/*1 | *1/*2 | 1.04% | 4.17% | 2.08% | 0 | 1.82% |
| *1/*7 | *1/*10 | *1/*1 | *1 /*15 | 0 | 0 | 0 | 1.04% | 0.26% |
| *1/*7 | *1/*10 | *1/*1 | *2/*2 | 1.04% | 0 | 0 | 1.04% | 0.52% |
| *1/*7 | *1/*10 | *1/*1 | *2/*3 | 0 | 1.04% | 0 | 0 | 0.26% |
| *1/*7 | *1/*10 | *1/*3 | *2/*2 | 0 | 1.04% | 0 | 0 | 0.26% |
| *1/*7 | *1/*10 | *1/*31 | *1/1 | 0 | 0 | 0 | 1.04% | 0.26% |
| *1/*7 | *1/*2 | *1/*1 | *1/1 | 0 | 0 | 1.04% | 0 | 0.26% |
| *1/*7 | *1/*5 | *1/*1 | *1/1 | 0 | 1.04% | 0 | 0 | 0.26% |
| *1/*7 | *10/*41 | *1/*1 | *1/1 | 3.13% | 0 | 1.04% | 0 | 1.04% |
| *1/*7 | *10/*41 | *1/*1 | *1/*2 | 1.04% | 0 | 0 | 0 | 0.26% |
| *1/*7 | *10/*10 | *1/*1 | *1/1 | 1.04% | 1.04% | 4.17% | 5.21% | 2.86% |
| *1/*7 | *10/*10 | *1/*1 | *1/*2 | 0 | 3.13% | 0 | 1.04% | 1.04% |
| *1/*7 | *10/*10 | *1/*1 | *1/*3 | 0 | 1.04% | 0 | 0 | 0.26% |
| *1/*7 | *10/*10 | *1/*1 | *1 /*15 | 0 | 0 | 0 | 1.04% | 0.26% |
| *1/*7 | *10/*10 | *1/*1 | *1/*17 | 0 | 1.04% | 0 | 0 | 0.26% |
| *1/*7 | *10/*10 | *1/*1 | *2/*2 | 0 | 2.08% | 0 | 0 | 0.52% |
| *1/*7 | *10/*10 | *1/*3 | *1/1 | 1.04% | 0 | 1.04% | 0 | 0.52% |
| *1/*7 | *10/*10 | *3/*3 | *1/1 | 0 | 1.04% | 0 | 0 | 0.26% |
| *1/*7 | *10/*14 | *1/*1 | *2/*15 | 0 | 1.04% | 0 | 0 | 0.26% |
| *1/*7 | *14/*14 | *1/*1 | *1/1 | 0 | 1.04% | 0 | 0 | 0.26% |
| *1/*7 | *2/*10 | *1/*1 | *1/1 | 0 | 1.04% | 0 | 0 | 0.26% |
| *1/*7 | *2/*10 | *1/*1 | *1/2 | 1.04% | 1.04% | 0 | 0 | 0.52% |
| *1/*7 | *2/*10 | *1/*1 | *1/*3 | 1.04% | 0 | 1.04% | 1.04% | 0.78% |
| *1/*7 | *2/*10 | *1/*1 | *2/*2 | 0 | 0 | 1.04% | 0 | 0.26% |
| *1/*7 | *2/*14 | *1/*8 | *1/1 | 0 | 0 | 1.04% | 0 | 0.26% |
| *1/*7 | *2/*2 | *1/*1 | *1/*17 | 0 | 0 | 1.04% | 0 | 0.26% |
| *1/*7 | *2/*2 | *1/*3 | *1/1 | 0 | 1.04% | 0 | 0 | 0.26% |
| *1/*7 | *2/*41 | *1/*1 | *1/1 | 0 | 0 | 1.04% | 0 | 0.26% |
| *1/*7 | *5/*10 | *1/*1 | *1/2 | 0 | 1.04% | 0 | 1.04% | 0.52% |
| *1/*7 | *5/*10 | *1/*1 | *2/*2 | 1.04% | 0 | 0 | 0 | 0.26% |
| *1/*7 | *5/*10 | *1/*1 | *2/*3 | 0 | 1.04% | 0 | 0 | 0.26% |
| *1/*7 | *5/*10 | *1/*1 | *2A/*17 | 0 | 1.04% | 0 | 0 | 0.26% |
| *1/*7 | 1*2/10 | *1/*1 | *1/2 | 0 | 1.04% | 0 | 0 | 0.26% |
| *2/*7 | *1/*10 | *1/*1 | *1/1 | 1.04% | 0 | 0 | 0 | 0.26% |
| *2/*7 | *10/*10 | *1/*1 | *2/*2 | 1.04% | 0 | 0 | 0 | 0.26% |
| *5/*7 | *1/*1 | *1/*1 | *1/*1 | 3.13% | 0 | 1.04% | 1.04% | 1.30% |
| *5/*7 | *1/*1 | *1/*1 | *1/*2 | 0 | 0 | 1.04% | 0 | 0.26% |
| *5/*7 | *1/*1 | *1/*1 | *2/*2 | 1.04% | 0 | 0 | 0 | 0.26% |
| *5/*7 | *1/*10 | *1/*1 | *1/*1 | 4.17% | 2.08% | 5.21% | 4.17% | 3.91% |
| *5/*7 | *1/*10 | *1/*1 | *1/*2 | 3.13% | 3.13% | 1.04% | 1.04% | 2.08% |
| *5/*7 | *1/*10 | *1/*1 | *2/*2 | 1.04% | 1.04% | 0 | 0 | 0.52% |
| *5/*7 | *1/*10 | *1/*3 | *1/*2 | 0 | 0 | 1.04% | 0 | 0.26% |
| *5/*7 | *1/*2 | *1/*1 | *1/*1 | 0 | 0 | 1.04% | 0 | 0.26% |
| *5/*7 | *1/*2 | *1/*1 | *1/*2 | 0 | 0 | 0 | 1.04% | 0.26% |
| *5/*7 | *1/*2 | *1/*1 | *1/*3 | 0 | 1.04% | 0 | 0 | 0.26% |
| *5/*7 | *1/*2 | *1/*3 | *1/*3 | 0 | 1.04% | 0 | 0 | 0.26% |
| *5/*7 | *1/*2 | *2/*3 | *1/*2 | 1.04% | 0 | 0 | 0 | 0.26% |
| *5/*7 | *10/*41 | *1/*1 | *1/*1 | 0 | 0 | 1.04% | 0 | 0.26% |
| *5/*7 | *10/*41 | *1/*1 | *1/*2 | 1.04% | 0 | 0 | 1.04% | 0.52% |
| *5/*7 | *10/*10 | *1/*1 | *1/*1 | 1.04% | 1.04% | 3.13% | 4.17% | 2.34% |
| *5/*7 | *10/*10 | *1/*1 | *1/*2 | 3.13% | 1.04% | 0 | 3.13% | 1.82% |
| *5/*7 | *10/*10 | *1/*1 | *1/*3 | 1.04% | 0 | 0 | 1.04% | 0.52% |
| *5/*7 | *10/*10 | *1/*1 | *2/*2 | 0 | 1.04% | 0 | 1.04% | 0.52% |
| *5/*7 | *10/*10 | *1/*1 | *2/*3 | 0 | 1.04% | 0 | 0 | 0.26% |
| *5/*7 | *10/*14 | *1/*1 | *1/*1 | 0 | 0 | 1.04% | 1.04% | 0.52% |
| *5/*7 | *10/*14 | *1/*1 | *1/*2 | 0 | 1.04% | 0 | 0 | 0.26% |
| *5/*7 | *14/*14 | *1/*1 | *1/*2 | 0 | 0 | 0 | 1.04% | 0.26% |
| *5/*7 | *2/*10 | *1/*1 | *1/*1 | 0 | 0 | 2.08% | 0 | 0.52% |
| *5/*7 | *2/*10 | *1/*1 | *1/*2 | 1.04% | 1.04% | 0 | 0 | 0.52% |
| *5/*7 | *2/*10 | *1/*1 | *1/*3 | 1.04% | 0 | 0 | 0 | 0.26% |
| *5/*7 | *2/*10 | *1/*1 | *2/*2 | 0 | 1.04% | 0 | 0 | 0.26% |
| *5/*7 | *2/*10 | *1/*1 | *2/*3 | 1.04% | 0 | 0 | 0 | 0.26% |
| *5/*7 | *2/*10 | *1/*3 | *1/*1 | 0 | 0 | 1.04% | 0 | 0.26% |
| *5/*7 | *2/*2 | *1/*1 | *1/*1 | 0 | 0 | 0 | 1.04% | 0.26% |
| *5/*7 | *2/*2 | *1/*1 | *1/*2 | 0 | 1.04% | 0 | 0 | 0.26% |
| *5/*7 | *2/*41 | *1/*1 | *1/*2 | 0 | 0 | 0 | 1.04% | 0.26% |
| *5/*7 | *5/*10 | *1/*1 | *1/*1 | 0 | 1.04% | 0 | 0 | 0.26% |
| *5/*7 | *5/*10 | *1/*1 | *1/*2 | 0 | 0 | 1.04% | 0 | 0.26% |
| *5/*7 | 1*2/10 | *1/*1 | *1/1 | 0 | 1.04% | 0 | 0 | 0.26% |
| *5/*7 | 1*2/2 | *1/*1 | *1/2 | 0 | 0 | 0 | 1.04% | 0.26% |
| *5/*7 | 10*2/10 | *1/*1 | *1/1 | 0 | 0 | 1.04% | 0 | 0.26% |
| *5/*7 | 10*2/10 | *1/*1 | *2/*2 | 0 | 0 | 0 | 1.04% | 0.26% |
| *5/*7 | 2*2/2 | *1/*8 | *1/2 | 0 | 0 | 1.04% | 0 | 0.26% |
| *5/*5 | *1/*1 | *1/*1 | *2/*2 | 0 | 0 | 0 | 1.04% | 0.26% |
| *5/*5 | *1/*10 | *1/*1 | *1/*1 | 3.13% | 0 | 0 | 0 | 0.78% |
| *5/*5 | *1/*10 | *1/*1 | *1/*2 | 1.04% | 0 | 0 | 0 | 0.26% |
| *5/*5 | *1/*10 | *1/*1 | *1/*3 | 0 | 1.04% | 0 | 0 | 0.26% |
| *5/*5 | *1/*10 | *1/*3 | *1/1 | 1.04% | 0 | 0 | 0 | 0.26% |
| *5/*5 | *1/*5 | *1/*1 | *1/2 | 1.04% | 0 | 0 | 0 | 0.26% |
| *5/*5 | *10/*10 | *1/*1 | *1/1 | 0 | 0 | 0 | 2.08% | 0.52% |
| *5/*5 | *10/*10 | *1/*1 | *1/*2 | 0 | 1.04% | 0 | 0 | 0.26% |
| *5/*5 | *10/*10 | *1/*1 | *2/*2 | 1.04% | 0 | 1.04% | 0 | 0.52% |
| *5/*5 | *41/*41 | *1/*1 | *1/*3 | 1.04% | 0 | 0 | 0 | 0.26% |
| *5/*5 | *5/*41 | *1/*1 | *1/*3 | 1.04% | 0 | 0 | 0 | 0.26% |
| *7/*7 | *1/*1 | *1/*1 | *1/*1 | 1.04% | 1.04% | 0 | 1.04% | 0.78% |
| *7/*7 | *1/*1 | *2/*8 | *1/*2 | 1.04% | 0 | 0 | 0 | 0.26% |
| *7/*7 | *1/*10 | *1/*1 | *1/*1 | 1.04% | 1.04% | 2.08% | 2.08% | 1.56% |
| *7/*7 | *1/*10 | *1/*1 | *1/*2 | 1.04% | 0 | 0 | 1.04% | 0.52% |
| *7/*7 | *1/*10 | *1/*1 | *2/*2 | 0 | 1.04% | 1.04% | 0 | 0.52% |
| *7/*7 | *1/*10 | *1/*3 | *1/*1 | 1.04% | 0 | 0 | 0 | 0.26% |
| *7/*7 | *1/*10 | *1/*3 | *1/*2 | 1.04% | 0 | 0 | 0 | 0.26% |
| *7/*7 | *1/*10 | *1/*8 | *1/1 | 1.04% | 0 | 0 | 0 | 0.26% |
| *7/*7 | *1/*41 | *1/*1 | *1/*2 | 0 | 1.04% | 0 | 0 | 0.26% |
| *7/*7 | *1/*41 | *1/*8 | *1/*1 | 0 | 0 | 1.04% | 0 | 0.26% |
| *7/*7 | *10/*41 | *1/*1 | *1/*2 | 0 | 1.04% | 0 | 0 | 0.26% |
| *7/*7 | *10/*10 | *1/*1 | *1/*1 | 1.04% | 2.08% | 2.08% | 2.08% | 1.82% |
| *7/*7 | *10/*10 | *1/*1 | *1/*17 | 0 | 0 | 0 | 1.04% | 0.26% |
| *7/*7 | *10/*10 | *1/*1 | *1/*2 | 1.04% | 0 | 0 | 3.13% | 1.04% |
| *7/*7 | *10/*10 | *1/*1 | *2/*2 | 1.04% | 0 | 0 | 2.08% | 0.78% |
| *7/*7 | *10/*10 | *1/*3 | *1/*1 | 1.04% | 1.04% | 0 | 1.04% | 0.78% |
| *7/*7 | *10/*10 | *1/*3 | *1/*17 | 0 | 0 | 1.04% | 0 | 0.26% |
| *7/*7 | *10/*10 | *1/*8 | *1/*1 | 0 | 0 | 2.08% | 0 | 0.52% |
| *7/*7 | *10/*10 | *1/*31 | *1/*1 | 0 | 0 | 2.08% | 0 | 0.52% |
| *7/*7 | *2/*10 | *1/*1 | *1/*3 | 0 | 0 | 0 | 1.04% | 0.26% |
| *7/*7 | *2/*10 | *1/*3 | *1/*1 | 0 | 0 | 0 | 1.04% | 0.26% |
| *7/*7 | *2/*2 | *1/*1 | *1/*1 | 0 | 0 | 0 | 1.04% | 0.26% |
| *7/*7 | 1*2/1 | *1/*1 | *1/*2 | 1.04% | 0 | 0 | 0 | 0.26% |
| total | | | | 100% | 100% | 100% | 100% | 100% |
